# Soil pollution by heavy metals correlates with levels of faecal glucocorticoid metabolites of a fossorial amphisbaenian reptile

**DOI:** 10.1093/conphys/coab085

**Published:** 2021-11-16

**Authors:** José Martín, Isabel Barja, Gonzalo Rodríguez-Ruiz, Pablo Recio, Luis V García

**Affiliations:** 1 Departamento de Ecología Evolutiva, Museo Nacional de Ciencias Naturales, CSIC, Madrid, Spain; 2 Etho-Physiology Group, Unidad de Zoología, Facultad de Biología, Universidad Autónoma de Madrid, Madrid, Spain; 3 Centro de Investigación en Biodiversidad y Cambio Global (CIBC-UAM), Universidad Autónoma de Madrid, Madrid, Spain; 4 Departamento de Biogeoquímica, Ecología Vegetal y Microbiana, Instituto de Recursos Naturales y Agrobiología de Sevilla, CSIC, Sevilla, Spain

**Keywords:** soil pollution, reptiles, heavy metals, fossorial, faecal corticosterone metabolites, Amphisbaenian

## Abstract

Soil degradation may have strong negative consequences for soil biodiversity, but these potential effects are understudied and poorly understood. Concentration of nesting seabirds may be a source of soil pollution by heavy metals, which are incorporated into the food chain and may have toxicological effects in vertebrates, especially in fossorial animals with low dispersal ability. We examined whether contamination by heavy metals, derived from seagull depositions, and other soil characteristics, may affect the levels of faecal glucocorticoid metabolites (as a potential indicator of physiological stress) of the fossorial amphisbaenian reptile *Trogonophis wiegmanni*. We found a relationship between soil pollution by heavy metals and increased levels of faecal corticosterone metabolite of the amphisbaenians that live buried in those soils. This can be due to the strong endocrine disruption effect of heavy metals. In addition, there was an independent effect of the soil texture, with amphisbaenians showing higher levels of faecal corticosterone metabolite in soils with less sand and more silt and clay, which are more energetically costly to dig. Long-term exposure to high glucocorticoid levels might have serious effects on health state and fitness of fossorial animals that may be unnoticed. Our study emphasizes that, to prevent future conservation problems, we need to perform periodic surveys on the physiological health state of the little-known subterranean biodiversity.

## Teaser

Soil pollution by heavy metals may negatively affect the health of animals living inside these soils. This study shows that populations of a fossorial burrowing reptile inhabiting more polluted habitats and soils more difficult to dig have higher levels of faecal corticosterone metabolite, likely reflecting higher physiological stress levels.

## Introduction

Soil degradation is one of the major environmental threats that can have serious consequences for conservation of soil biodiversity (Osman, 2013); Dominguez *et al.*, 2017; Tibbett *et al.*, 2020). This is important because soil biodiversity is considered to be a main factor in regulating the functioning of terrestrial ecosystems (Copley, 2000; Wolters, 2001; Bardgett and van der Putten, 2014), but this importance is often not appreciated (Decaens *et al.*, 2006; Tibbett *et al.*, 2020), and the absence of concern about fossorial animals from conservationists is conspicuous (Wolters, 2001; Böhm *et al.*, 2013).

Soil alteration and pollution may be due to the direct action of man, whose activities have led to profound modifications of the original soil horizons and the addition of residues and contaminants (Craul, 1992; Larramendy, 2016; Duarte *et al.*, 2017). However, there are also indirect causes of soil pollution. Among them, for example, the concentration of seabirds in nesting and resting areas induces profound changes in soil chemical properties, including eutrophication, salinization, acidification and nutrient imbalances (García *et al.*, 2002a,b). Moreover, seabirds may be the main vectors moving heavy metals and other pollutants from anthropic contaminated food sources to the soils (Headley, 1996; Otero, 1998; García *et al.*, 2002a,b; Blais *et al.*, 2005). Heavy metals can be, then, transferred from the soil to plants and invertebrates and go up the food chain (Walker *et al.*, 2012) to reach high toxicological concentrations in tissues of vertebrates such as reptiles (Loumbourdis, 1997; Campbell and Campbell, 2001; Márquez-Ferrando *et al.*, 2009; Grillitsch and Schiesari, 2010) or birds (Franceschini *et al.*, 2009; Manjula *et al.*, 2015; Orłowski *et al.*, 2015).

Soil pollution could have especially strong negative consequences for fossorial animals. These include many invertebrate species, but also some vertebrates that are highly specialized in an underground lifestyle. Up to 28% of the reptiles of the world (i.e. more than 2000 species), including many skinks, legless lizards, blind snakes and amphisbaenians are fossorial and spend all or most of their lives underground (Measey, 2006). However, their biology, ecology and conservation status is much less known than those of their epigeal relatives (Böhm *et al.*, 2013), probably because of the difficulty to study them (Measey, 2006; Henderson *et al.*, 2016). Research of fossorial animals is, however, important because they face different ecological challenges and threats than epigeal species and have evolved very peculiar adaptations to the underground life (Gans, 1974, 1978). Moreover, fossorial species may be at particular risk of extinction because their low dispersal ability (Martín *et al.*, 2021b) may preclude them to avoid local soil disturbances. Ignorance of these effects may result in a bad conservation state that may be going unnoticed (How and Shine, 1999; Decaens *et al.*, 2006; Measey *et al.*, 2009).

Amphisbaenians is one of the more notorious, but little conspicuous and understudied, groups of fossorial reptiles (Gans, 1978, 2005). Amphisbaenians have very specialized morphological and functional adaptations to a fossorial life, such as reduced vision, elongated body or loss of limbs (Gans, 1978, 2005; Navas *et al.*, 2004), which, however, constrain many other aspects of their ecology, such as habitat or diet selection or some reproductive parameters (Papenfuss, 1982; Colli and Zamboni, 1999; Webb *et al.*, 2000; Andrade *et al.*, 2006; Gomes *et al.*, 2009). Because amphisbaenians spent all their lives underground and often show high site fidelity (Martín *et al.*, 2021b), they must be especially affected by any soil alteration. In fact, natural and anthropogenic soil alterations seem to affect the body condition of amphisbaenians (Martín *et al.*, 2015, 2017). However, the processes implied in these negative effects of soil degradation and the consequences for health and conservation state of populations are understudied and not understood.

Conservation biologists are increasingly confronted with the need to provide conservation managers with data on the causal mechanisms underlying conservation problems—for example, to understand the factors affecting the health state of individuals, which may strongly affect the decline of populations and extinction of species. In this sense, in order for conservation strategies to be successful, it is important to understand the physiological responses of organisms to changes in their environment (Wikelski and Cooke, 2006; Angelier and Wingfield, 2013; Cooke *et al.*, 2013). In this context, it can be very useful to the study physiological stress responses of animals (Broom and Johnson, 1993; Wingfield *et al.*, 1997; Angelier and Wingfield, 2013). When an animal is subjected to a stressor, the endocrine stress response enhances the activation of the hypothalamic–pituitary–adrenocortical axis, stimulating the secretion of glucocorticoids (GCs) (corticosterone or cortisol, depending on the species) (Sapolsky *et al*., 2000; Melmed and Kleinberg, 2003). An increase of GC has different effects depending on the time of exposure to the stressor. Thereby, short-term stress has been related to an adaptive response for improving an individual’s fitness (Sapolsky *et al*., 2000; Wingfield and Romero, 2001), whereas long-term GC secretion may cause some important pathologies (e.g. reproductive failure, endocrine disruption, suppression of the immune system and/or gastrointestinal ulcerations) (Stewart, 2003; Romero, 2004). Physiological stress response plays a key role in the adaptability of animals to changes in the environment, as well as being a decisive factor in the stability of homeostasis (Möstl and Palme, 2002).

Levels of GC have been used as indicators of animal stress in several species of vertebrates including reptiles (e.g. Moore and Jessop, 2003; Tokarz and Summers, 2011; Kechnebbou *et al*., 2019; Megía-Palma *et al*., 2020, 2022). These GC levels can be measured not only in plasma (Romero, 2002; Bauer *et al*., 2008), but also in urine, faeces, hair, feathers, etc. (Teskey-Gerstl *et al*., 2000; Bosson *et al*., 2009; Tarjuelo *et al*., 2015). However, despite its wide use, mainly in the past (Broom and Johnson, 1993), the measurements of GC levels in plasma are influenced by the sampling conditions. This is because obtaining blood samples requires the capture, handling and puncture of the animal, which induce per se the activation of physiological stress responses, with GC levels varying depending on the handling time of the animals (Place and Kenagy, 2000; Young *et al*., 2004; Barja, 2015). Furthermore, the GC levels in plasma show variations due to the pulsatile secretion of hormones and the influence of the circadian cycle (Van Cauter *et al*., 1996). In contrast, the analysis of faecal GC metabolites is a non-invasive method, which minimizes the disturbances associated with the collection of blood samples (Barja *et al*., 2012). Nevertheless, the main disadvantages of this method are the difficulty to collect a large number of fresh faecal samples, mainly in animals with wide territories, and, for small animals, not getting the enough amount of individual faecal sample required for the analysis. Also, faecal samples lose GCs after a long storage time at −20°C between collection and analysis (Barja *et al*., 2012).

Particularly in wildlife studies, faecal cortisol/corticosterone metabolites have been widely used as a suitable non-invasive measure of the GC levels to evaluate responses during stressful circumstances (Barja *et al*., 2012; Barja, 2015), reflecting free GC in plasma and yielding an accurate profile of the adrenocortical activity (Touma and Palme, 2005). Multiple factors, such as human disturbances (Piñeiro *et al*., 2012; Navarro-Castilla *et al*., 2014a,b; Kechnebbou *et al*., 2019) or habitat type and seasonality (Hik *et al*., 2001; Huber *et al*., 2003; Sheriff *et al*., 2012), have been reported to influence the vertebrates’ physiological stress response in different ways decreasing the energy available for individuals to cope with environmental stressors (Selye, 1960).

Here, we aimed to examine whether soil pollution by heavy metals, derived from seagull depositions, may affect levels of faecal GC metabolites of fossorial reptiles. For this, we designed a field study in a North African island population of *Trogonophis wiegmanni* amphisbaenians, using the levels of corticosterone metabolites in faecal pellets of a large sample of individuals. We, then, related these faecal GC metabolites levels to the degree of heavy metal soil pollution at the capture sites. As heavy metals can be powerful endocrine disruptors and result in abnormal circulating hormone levels (Giesy *et al.*, 2003; Ottinger *et al.*, 2005; Meillère *et al.*, 2016), we predicted that soils with high contamination by heavy metals should induce higher levels of faecal GC metabolites to amphisbaenians living inside these soils. Nevertheless, we also examined the relationships with other soil physical and chemical characteristics to discard the possibility that a supposed effect of heavy metals was confounded with the effect of other soil properties that might also affect faecal GC metabolites levels of amphisbaenians.

## Methods

### Study area

We carried out the field study during 2018 and 2019 at the small archipelago of Chafarinas Islands (Spain), located in the southwestern area of the Mediterranean Sea (35°11’ N, 02° 25’ W), 2.5 nautical miles off the northern Moroccan coast (Ras el Ma, Morocco). It consists of three islands: Congreso (25.6 ha), Isabel II (15.1 ha) and Rey Francisco (13.9 ha). The aridity of the warm Mediterranean climate (an average annual precipitation of 300 mm) and the high soil salinity highly determine the type of vegetation, which is dominated by woody bushes (*Salsola*, *Suaeda*, *Lycium* and *Atriplex*) adapted to salinity and drought (García *et al.*, 2002a,b). Soils are poorly developed and immature and are characterized by a thin layer, rich in organic matter, which is underlain by the original volcanic rock (Clemente *et al.*, 1999; García, 2005; García *et al.*, 2007a). Two of the islands, Rey and Congreso, are uninhabited. These two islands support large nesting colonies of two species of seagulls; the Audouin’s gull (*Larus audouinii*) (221 nests in Rey in the year 2018), a rare and protected Mediterranean seabird, and the widespread yellow-legged gull (*Larus cachinnans*), a very abundant Mediterranean seabird (2380 nests in Rey and 6074 nests in Congreso in 2018; unpublished data of the Spanish National Parks authority). The population of yellow-legged gulls, which also uses the islands as a resting area all year round, has increased considerably in the past years (e.g. in 1976 the figures were around 300 nests in Rey and 600 nests in Congreso). The third island (Isabel) is inhabited since 1848 by a small human population (less than 50 people nowadays) and has had relatively lower seabird influence, although in the past years there is a new colony of Audouin’s gull in a rocky cliff area (223 nests in 2018) and groups of yellow-legged gulls are always resting along the island and are doing nesting attempts.

Previous studies have shown the negative influence of these gulls on the soil chemical properties of these islands (García *et al.*, 2002a,b; Martín *et al.*, 2015). Also, other studies examined, using isotopic markers (d15N), the concentration of heavy metals in faeces of gulls and in the soil along a gradient of seagull influence (García *et al.*, 2007b). Results showed much higher concentrations of heavy metals in gull faeces than in non-polluted soils (from 4× to 300×) and higher concentrations of most of EDTA extractable heavy metals in polluted soils (García *et al.*, 2007b). Both micronutrients and potentially toxic heavy metals (e.g. Cd, Ni or Cr) increased significantly in soils with clear seagull influence. Some micronutrients, such as Zn, may even exceed the established toxicity threshold in polluted soils (García *et al.*, 2002b).

### Soil characteristics

To characterize the physical and chemical characteristics of the soil in the study area, we made a series of random transects covering the whole area potentially used by amphisbaenians (excluding bare rocky areas without soil) and selected a total of 79 random points in the three islands. At each sampling point, geolocalized with a GPS, we took a bulked soil sample (~300 g) by mixing three subsamples taken under nearby stones like those used by amphisbaenians (Civantos *et al.*, 2003). We dug between the surface and up to 10 cm in depth (or less if the soil was less deep), coinciding with the soil layers used by amphisbaenians (J. Martín *et al.*, unpublished data). In the laboratory, soil samples were air-dried, crushed and sieved (<2 mm). Particles between 2 and 60 mm were weighed to calculate the percentage of ‘gravel’ in the sample. Thereafter, we used wet sieving to separate the ‘sand’ fraction (2–0.05 mm). The percentage of ‘silt’ (0.05–0.002 mm) and ‘clay’ (<0.002 mm) in the fine earth was determined by using the Bouyoucos hydrometer method (for details of physical analyses, see Dane and Topp, 2002).

We also used these soil samples to analyse chemical characteristics (for details see, Sparks, 1996). Briefly, we used a pressure-calcimeter to measure soil ‘total inorganic carbonates’. We used a modified Walkley and Black method to determine ‘organic carbon’ (C) and a Kjeldahl digestion and distillation titration of the produced ammonium to measure total ‘organic nitrogen’ (N). We extracted available ‘phosphorus’ (P) using sodium bicarbonate (0.5 M, pH 8.5) and measured it by visible spectrophotometry using ammonium molybdate and ascorbic acid. We used a combined electrode in soil paste with water or KCl (1:2.5) to measure ‘pH’. We also measured ‘electrical conductivity’ (EC) electrometrically in aqueous extracts (1:5 soil:water). In these extracts, we determined eight ‘soluble ions’ (Na^+^, K^+^, Ca^2+^, Mg^2+^, Cl^−^, NO_3_^−^, NH_4_^+^ and SO_4_^2−^); and we measured Na^+^ and K^+^ by flame photometry, Ca^2+^ and Mg^2+^ by atomic absorption spectroscopy, Cl^−^ by titration with AgNO_3_ and NO_3_^−^, NH_4_^+^ and SO_4_^2−^ by visible spectrophotometry. Finally, we determined the concentration of nine ‘heavy metals’ (Cd, Co, Cr, Cu, Fe, Mn, Ni, Pb and Zn) using inductively coupled plasma optical emission spectrometry (ICP-OES; Varian ICP 720-ES; Varian, Palo Alto, CA, USA), after extraction with a neutral 0.05 M-EDTA solution. This procedure estimates the bioavailable fraction of heavy metals, providing a better picture of the actual risk of soil heavy metals for living organisms (Gleyzes *et al.*, 2002).

Descriptive statistics of the variation of characteristics of soils available in the study area, as well as the effects of seagull influence on soils and vegetation, have been examined in previous soil surveys (García *et al.*, 2002a,b; Martín *et al.*, 2013a, 2015).

### Study species

The amphisbaenian *T. wiegmanni* is a Northwest African species found from Morocco to northeast Tunisia (Bons and Geniez, 1996). Similarly to other amphisbaenians, the knowledge of its ecology is limited, but there are increasing knowledge on its habitat use (Civantos *et al.*, 2003; Martín *et al.*, 2013a, 2021a), diet and prey detection (Bons and Saint Girons, 1963; Martín *et al.*, 2013b; López *et al.*, 2014; Baeckens *et al.*, 2017), thermal biology (Gatten and McClung, 1981; López *et al.*, 2002), reproduction (Bons and Saint Girons, 1963) and population and social biology (Martín *et al.*, 2011a,b, 2012, 2020, 2021b). This species is listed as of ‘Least Concern’ by the IUCN in view of its wide distribution and ‘presumed’ large population (Mateo *et al.*, 2009). Two previous studies showed that natural soil salinization and compaction of the soil affected negatively to body condition indexes of amphisbaenians (Martín *et al.*, 2015, 2017). However, most of the potential threats, especially soil pollution, to this and other amphisbaenian species have been largely understudied and are, therefore, not well known.

### Amphisbaenian sampling procedures

We visited the Chafarinas islands during four field campaigns of 2 weeks duration each in spring (March–April), coinciding with the breeding season of amphisbaenians, and autumn (September–October) 2018 and 2019. In each campaign, we haphazardly followed different routes covering all the available habitats used by amphisbaenians in the three islands (Martín *et al.*, 2011c; García-Roa *et al.*, 2014). During the day between 07:00 and 18:00 (GMT), we searched for amphisbaenians by lifting almost all rocks and stones found, which were enough (>40% of rock surface cover) to allow a large effective survey area. Amphisbaenians were abundant and easy to find under rocks (Martín *et al.*, 2011c, 2013a). We captured live amphisbaenians by hand and collected their faeces in the field. Amphisbaenians usually defecate most gastrointestinal contents when handled, but, if needed, we also compressed gently their vents for a few seconds to force the expulsion of faeces. If an amphisbaenian did not excrete faeces in a short time, we stopped further handling to avoid disturbance. We only used samples from adult individuals. We individually stored faeces in Eppendorf vials that were kept inside a portable refrigerator with ice in the field, and a few hours later stored at −20°C in a freezer at the Chafarinas Field Station.

In the field, for each individual, we measured total length (from the tip of the snout to the tip of the tail) with a metallic ruler (to the nearest 1 mm) and body mass with a pesola spring scale (to the nearest 0.01 g). To avoid confounding effects, we measured individuals with empty stomachs after extracting the faecal samples. We used as a body condition index the residuals of an ordinary least squares linear regression of log-transformed mass against log-transformed total length (lineal regression, *r* = 0.90, *F*_1,463_ = 2048.58, *P* < 0.0001). Such residuals are considered to provide the cleanest way to separate the effects of condition from the effects of body size, and are used as proxies of health state in many animals (Green, 2000; Brischoux *et al.*, 2012) including this amphisbaenian (Martín *et al.*, 2015, 2017).

We also determined sexes of amphisbaenians by examining the presence of hemipenis in the cloacae (Martín *et al.*, 2011b). Amphisbaenians were individually marked with Passive Integrated Transponders (PIT) tags as a part of a population monitoring study (Recio *et al.*, 2019), which also allowed us to avoid repeated sampling of the same individual. We released amphisbaenians at their exact point of capture in <5 min after finding them. Location of capture points were measured with a GPS (GPSmap 60CSx; Garmin Ltd, Olathe, KS, USA).

We aimed to obtain faecal samples of amphisbaenians (*n* = 465) that were as evenly as possible distributed between sexes (56.3% females vs. 43.7% males), seasons (57.0% spring vs. 43.0% autumn) and years (57.2% in 2018 vs. 42.8% in 2019). However, although we made a similar searching effort in all the surface of the three islands, the number of samples per island (3.5% Congreso vs. 46.0% Rey vs. 50.5% Isabel) was highly dependent on the actual abundances of amphisbaenians in each island (Martín *et al.*, 2011c). Nevertheless, these different abundances between islands seem to be a consequence of differences in the availability of appropriate microhabitats for amphisbaenians rather than to differences in population densities in the occupied sites (J. Martín, unpublished data).

Captures and observations were performed under licence by the Organismo Autónomo de Parques Nacionales (Spain). All applicable international, national and institutional (Consejo Superior de Investigaciones Científicas) guidelines for the care and use of animals were followed. Research procedures were approved by the Comisión Ética de Experimentación Animal (CEEA) of the Museo Nacional de Ciencias Naturales, CSIC.

### Extraction and quantification of faecal corticosterone metabolites

At the end of each field campaign, we transported within a few hours all faecal samples in a portable freezer with ice to the Ethology and Endocrinology Laboratory of the Autonomous University of Madrid for the quantification of faecal GC metabolites levels. From collection of samples and until analyses, we kept the cold chain since the temperature seems to affect GC levels (Navarro-Castilla *et al.*, 2014a,b). Also, to avoid that GC levels decreased with storage time (Barja *et al*., 2012), all samples were frozen for a similar time, always less than 1 month, before analyses.

To extract faecal corticosterone metabolites in faces of amphisbaenians, the frozen faecal samples were first pulverized and dried in an oven at 90°C for 3–4 h. Once dry, 0.05 g of each sample was weighed and 500 μl of phosphate buffer saline (PBS) and 500 μl of 100% methanol were added to an eppendorf tube. The tubes were agitated using a vortex shaker and later transferred to an orbital shaker, where they spent 16 h to homogenize the sample and the chemicals. To finish the extraction, the extract resulting from the orbital shaker was centrifuged at 2500 rpm for 15 min and the supernatant was transferred to polyurethane tubes, suitable for the preservation of hormones, which were kept at −20°C until they were quantified.

Quantification of corticosterone metabolites in undiluted faecal extracts was performed in the first week after extraction with a commercial enzyme immunoassay kit (D-24145; Demeditec Diagnostics GmbH, Kiel, Germany). The evaluation of the levels of corticosterone metabolites in the faecal extracts (expressed in ng/g dry excrement) was done in a spectrophotometer (Microplate Reader, MR 600; Dynatech Industries Pvt. Ltd, Bangalore, India). All faecal samples were analysed randomly in the assays and faecal extracts were analysed in duplicates.

The enzyme immunoassays with DEMEDITEC-kits were successfully validated by carrying out the corresponding parallelism, accuracy and precision tests. Parallel displacement curves were obtained by comparing serial dilutions (1:16, 1:8, 1:4, 1:2, 1:1) of pooled faecal extracts with the standard curve provided by the manufacturer (*R^2^* = 0.94, *P* > 0.05). The percentage of recovery (accuracy) of the corticosterone was above 90% in the samples and, therefore, the extracts did not contain substances that interfered with the quantification. Precision was tested through intra- and inter- assay coefficients of variation for faecal samples, being 9.6% (*N* = 98) and 11.9% (*N* = 15), respectively. The assay sensitivity for corticosterone was >4.1 ng/ml. These results clearly supported that the used kits were correctly measuring corticosterone metabolite concentrations in the collected samples without specifically requiring an ACTH test. Moreover, we did not perform an ACTH challenge test to show the time passed for peak concentration in faeces because the study’s aim was to consider the variation in faecal corticosterone metabolite levels due to a continuous stressor.

### Data analysis

Because we did not collect a soil sample for each individual amphisbaenian, we referred the GPS location of each capture site to the GPS location of the nearest soil sampling point (distance between capture and soil sample point, mean ± SE = 19.8 ± 2.5 m; range = 3–29 m). This was justified because soil characteristics were very similarly homogeneous in an area of such dimensions surrounding the soil sampling point, and because amphisbaenians have a high site fidelity and move over very small areas (approx. between 1 and 25 m^2^; Martín *et al.*, 2021b). Therefore, we considered that each soil sampling point reflected the soil environment long-term experienced by one individual captured in that area. We designated each of these soil sampling points as a block, and since these points were selected randomly, then, we considered our sampling as a randomized complete block design. Thus, each of the samples of amphisbaenians could be considered as replicates, capturing all the variance of the response variable (faecal corticosterone metabolite levels). Given the unequal distribution and densities of amphisbaenians in the islands (Martín *et al.*, 2011c), we collected valid information of amphisbaenians related to 33 of the soil sampling points (mean ± SE = 14 ± 1 individuals/point, range = 1–82 indiv./point).

We first used a Lineal Mixed Model (LMM) to estimate variation in individual faecal corticosterone metabolites levels (log10 transformed) of adult amphisbaenians as the response variable, depending on their sex (male vs. female), season (spring vs. autumn) and year (2018 vs. 2019) as categorical fixed factors, and included the soil sampling point as a random factor. Residuals of the model fulfilled the normality and homoscedasticity assumptions (tested with Shapiro–Wilk’s and Levene’s tests, respectively).

We then explored which soil variables might better explain variations in levels of faecal corticosterone metabolite of amphisbaenians. First, given the high correlation between some of the 28 variables that characterized the physical and chemical properties of the soils, we used a principal component analysis (PCA) with a varimax rotation to synthesize all variables into a small number of components that described the main axis of variation in soil characteristics. This PCA was then used to identify the variables that better described each of these axes (those with the highest loadings; see Results).

Thereafter, we used a multimodel inference (Anderson, 2008) and made a set of candidate models using Generalized Linear Models (GLZ), with a normal distributional and a log link function, the levels of faecal corticosterone metabolite (log_10_-transformed) as the response variable, all the combinations of the previously selected, more explicative, soil variables (see results) as continuous covariates and the sampling year as a factor. We calculated Akaike values (AICc) and those candidate models with ΔAICc ≤ 4 were considered sufficiently informative (Anderson, 2008). We used the Likelihood ratio test as the omnibus test to prove that a model was different from the null model. After selecting the best final model (i.e. the one with the lowest AICc value), we conducted log-likelihood ratio χ^2^tests and Wald’s χ^2^tests to estimate the significance of the contributing variables. We also checked that residuals of the final model fulfilled the normality and homoscedasticity assumptions. Extracted residuals of the model and partial predicted values of the response variable (corticosterone metabolite) were used to made partial residual plots for the soil variables included in the final model (Moya-Laraño and Corcobado, 2008). Statistical analyses were made with the Statistica 8.0 software (StatSoft Inc. Tulsa, OK, USA).

## Results

### Soil characteristics

Physical and chemical characteristics of the soils where we found amphisbaenians are summarized n Table 1. The PCA for variables describing soil characteristics produced four principal components (PCs) with eigenvalues greater than two, which together accounted for 71.2% of the total variance (Table 1). The first PC (PC-1) was positively highly correlated with the concentration of eight of the nine heavy metals (all but Pb) and negatively correlated with soil pH. Thus, soils with higher PC-1 scores were more acidic and had higher concentrations of most of the heavy metals, except Pb. The second PC (PC-2) described a gradient of soils with increasing values of extractable P and some soluble ions (NO_3_^−^, NH_4_^+^, SO_4_^2−^ and Ca^2+^) and EC (i.e. increased salinity). Thus, high values in this component corresponded to highly eutrophicated soils. The third PC (PC-3) described a gradient of soil texture, from sandy soils to soils with more silt and clay. Finally, the fourth PC (PC-4) described a marine-dependent salinity gradient towards soils enriched in salt of marine origin (i.e. dominated by Cl^−^, Na^+^ and Mg^2+^).

**Table 1:** Physical and chemical characteristics (mean **±** SE; range) of soils used by *T wiegmanni* amphisbaenians in the study area and correlations of the variables with the PCs from a PCA for these soil variables

	Mean ± SE	Range	PC-1	PC-2	PC-3	PC-4
Gravel (%)	37.8 **±** 1.8	10.7–50.8	−0.45	0.14	0.56	−0.28
Sand (%)	61.6 **±** 2.0	40.9–83.9	−0.07	0.02	**−0.88**	−0.24
Silt (%)	15.4 **±** 1.0	5.3–24.9	0.24	−0.09	**0.67**	−0.22
Clay (%)	20.0 **±** 1.5	6.4–36.8	−0.03	0.03	**0.76**	0.43
pH _H2O (1/2.5)_	7.69 **±** 0.14	5.40–8.58	**−0.75**	−0.18	−0.14	−0.09
pH _KCl (1/2.5)_	7.14 **±** 0.12	5.15–7.89	**−0.79**	−0.30	−0.14	−0.07
Total inorganic carbonates (%)	10.8 **±** 2.1	0.8–39.7	−0.35	−0.04	−0.49	−0.35
Organic C (%)	2.9 **±** 0.3	0.7–7.2	0.29	0.08	0.12	0.14
N _Kjeldalh_ (%)	0.3 **±** 0.1	0.1–1.1	0.41	0.33	0.12	0.20
P _Olsen_ (mg kg^−1^ soil)	194.9 **±** 36.2	16.7–750.0	0.18	**0.91**	−0.02	0.07
Electrical conductivity (dS m^−1^)	0.52 **±** 0.07	0.16–1.78	0.16	**0.76**	0.01	0.49
Na^+^_1/5_ (mg kg^−1^ soil)	197.3 **±** 21.6	58.0–476.7	0.06	0.21	0.06	**0.68**
K^+^_1/5_ (mg kg^−1^ soil)	146.2 **±** 22.9	21.9–498.4	0.41	0.53	0.15	0.55
Ca^2+^_1/5_ (mg kg^−1^ soil)	155.6 **±** 13.1	59.2–403.4	0.12	**0.73**	−0.01	0.42
Mg^2+^_1/5_ (mg kg^−1^ soil)	57.8 **±** 8.6	19.3–248.5	−0.04	0.30	0.06	**0.79**
Cl^−^_1/5_ (mg kg^−1^ soil)	551.0 + 92.9	75.8–1992.5	−0.01	0.08	0.10	**0.89**
NO_3_^−^ (mg kg^−1^ soil)	20.4 **±** 3.4	2.2–55.0	0.13	**0.86**	0.20	0.09
NH_4_^+^ (mg kg^−1^ soil)	74.2 **±** 18.5	13.9–370.0	0.10	**0.91**	−0.03	0.07
SO_4_^2−^_1/5_ (mg kg^−1^ soil)	84.4 **±** 18.6	0.0–378.4	0.07	**0.80**	−0.09	−0.09
Cd (mg kg^−1^ soil)	0.17 **±** 0.02	0.06–0.46	**0.85**	−0.03	0.23	0.06
Co (mg kg^−1^ soil)	0.31 **±** 0.05	0.09–1.19	0.50	0.02	**0.69**	−0.06
Cr (mg kg^−1^ soil)	0.20 **±** 0.02	0.07–0.52	**0.90**	0.13	−0.09	−0.06
Cu (mg kg^−1^ soil)	4.67 **±** 0.67	1.31–18.18	**0.82**	−0.02	−0.15	0.04
Fe (mg kg^−1^ soil)	78.40 **±** 16.37	12.54–401.38	**0.90**	0.30	0.14	0.06
Mn (mg kg^−1^ soil)	62.39 **±** 12.26	11.83–283.01	**0.78**	0.00	0.51	0.01
Ni (mg kg^−1^ soil)	0.48 **±** 0.06	0.21–1.63	0.52	0.08	**0.62**	−0.16
Pb (mg kg^−1^ soil)	24.09 **±** 2.59	10.95–79.12	0.29	−0.15	−0.54	−0.04
Zn (mg kg^−1^ soil)	36.14 **±** 6.44	4.94–130.80	**0.90**	0.19	−0.04	0.03
						
Eigenvalue			9.56	4.61	3.63	2.14
% Variance explained			34.2	16.4	12.9	7.7

Factor loadings >0.60 are captured in boldface.

Using these PCA results, we selected the soil variables that may better describe the four identified axis of variation of soil characteristics in the study area (i.e. those variables with the highest loadings; see Table 1). For the first axis describing concentrations of heavy metals, most heavy metals showed high loadings (between 0.50 and 0.90) and strong correlations between them. Thus, we could not confidently select any particular metal as the most representative. We instead calculated the total metal load, as the sum of the concentrations of eight heavy metals, all but Pb that seems to be unrelated to the others. For the second axis describing eutrophication, we selected concentrations of P (loading = 0.91). Representing the third axis of soil texture, we selected the percentage of sand (loading = −0.88). Finally, for the fourth axis of marine salinity, we selected Cl^−^ concentration (loading = 0.89).

### Levels of faecal corticosterone metabolite of individual amphisbaenians

**Figure 1:**
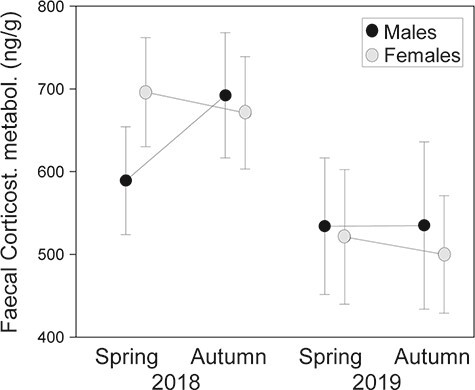
Sex, seasonal and interannual variation in mean (± SE) levels of faecal corticosterone metabolite (ng/g dry excrement) of individual *T. wiegmanni* amphisbaenians

Individual levels of faecal corticosterone metabolites of amphisbaenians (Fig. 1) did not significantly differ between sexes (LMM, *F*_1,425_ = 0.31, *P* = 0.58) or seasons (*F*_1,425_ = 1.35, *P* = 0.25), but overall values varied significantly between years (*F*_1,425_ = 37.42, *P* < 0.0001), being higher in 2018 than in 2019. All the two-way and the three-way interactions were not significant (sex × season: *F*_1,425_ = 0.04, *P* = 0.85; sex × year: *F*_1,425_ = 0.34, *P* = 0.56; season × year: *F*_1,425_ = 2.18, *P* = 0.14; sex × season x year: *F*_1,425_ = 1.07, *P* = 0.30) (Fig. 1). In addition, there were also significant differences in faecal corticosterone metabolites levels among sampling points (random factor, *F*_32,425_ = 2.54, *P* < 0.0001).

Levels of faecal corticosterone metabolites of amphisbaenians were not significantly related to body condition indexes (lineal regression, *r* = −0.02, *F*_1,463_ = 0.16, *P* = 0.69).

### Relationships between soil characteristics and levels of faecal corticosterone metabolite of amphisbaenians

**Figure 2:**
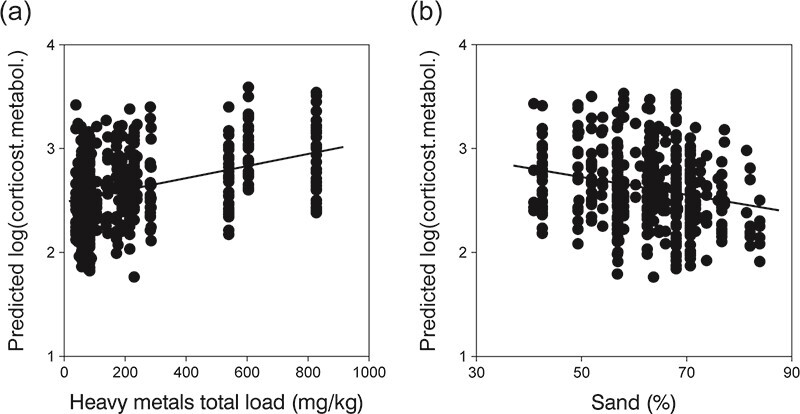
Partial residual plots extracted from the final GLZ model showing the relationship between (**a**) total load of heavy metals (all but Pb) (mg/kg soil) or (**b**) percentage of sand at a soil sampling point and the partial predicted levels of faecal corticosterone metabolite (log_10_-transformed ng/g) in *T. wiegmanni* amphisbaenians found surrounding that point.

After multimodel inference (see Supplementary Table S1), the final selected model, the one with the lowest AICc value, suggested that the total load of soil heavy metals, but also the percentage of sand in the soil and the sampling year, could predict the variations in levels of corticosterone metabolite in faeces of amphisbaenians (Table 2). Therefore, amphisbaenians inhabiting soils with higher concentrations of heavy metals or soils with less sand showed higher levels of faecal corticosterone metabolite (Fig. 2), but these levels also varied depending on the sampling year.

**Table 2:** Final GLZ model, selected by multimodel inference (see Supplementary Table S1), for the effects of soil characteristics and sampling year on the levels of faecal corticosterone metabolite (log_10_ transformed)

	Log-likelihood	*χ^2^*	*P*	Estimate ± SE	Wald’s *χ^2^*	*P*
Intercept	−200.48			1.0053 ± 0.0523	369.31	< 0.0001
Total load of heavy metals	−196.16	8.97	0.0027	0.0001 ± 0.0001	12.87	0.0003
Sand	−200.64	9.67	0.0019	−0.0012 ± 0.0008	2.54	0.11
Year	−180.33	31.65	< 0.0001	0.0373 ± 0.0065	32.39	< 0.0001

Results of log-likelihood ratio *χ*^2^ tests, estimates (± SE) and Wald’s *χ*^2^ tests of the variables included in the model are shown.

## Discussion

The results of this study strongly suggest that there was a direct relationship between contamination by heavy metals in polluted areas and the increase in faecal corticosterone metabolite levels of the *T. wiegmanni* amphisbaenians that live in those soils. In addition, there was an additional effect of the soil texture, such that amphisbaenians had more corticosterone metabolite levels in fine-textured heavy soils (i.e. with less sand and more silt and clay), which are presumably more energetically costly to dig. Therefore, these results suggest that the soil characteristics and its conservation state may greatly affect to the health state of fossorial vertebrates.

We firstly found that the concentrations of heavy metals in the soil, which in these islands have been shown to be significantly dependent on the abundance of seagulls (García *et al.*, 2002a,b, 2007b; Martín *et al.*, 2015), are related to faecal corticosterone metabolite levels of amphisbaenians. Many studies, mainly laboratory experiments, have shown that trace elements can have a strong endocrine disruption effect, which produced abnormal or modified levels of circulating hormones (Colborn *et al.*, 1993; Giesy *et al.*, 2003; Ottinger *et al.*, 2005; Tan *et al.*, 2009). For example, there is a relationship between heavy metals in blood and corticosterone levels in plasma of several wild bird species (Meillère *et al.*, 2016; Franceschini *et al.*, 2009, 2017). We do not have data of heavy metals concentrations in blood of amphisbaenians. However, reptiles are able in polluted habitats to transfer trace elements from the peripheral circulation to the growing skin where they are accumulated and later expelled when the skin is sloughed (Hopkins *et al.*, 2001; Jones and Holladay, 2006). In this regard, we found that levels of heavy metals in the shed skin of *T. wiegmanni* amphisbaenians from sites with more soil contamination are until 2–3 times higher than in other less polluted areas (Martín *et al.*, in press), suggesting a relationship between soil pollution and levels of heavy metals inside the bodies of amphisbaenian.

One particular case was found for Pb, which concentrations seemed unrelated to the other heavy metals measured. In our study area, this element was shown to be ~50% relatively less abundant in seabird affected soils that in other areas (Martín *et al.*, 2015). This is mainly explained because Pb was rather more abundant in the soils of the inhabited Isabel island as a result of direct human contamination from water dissolution of old lead pipes, paint chips from whitewashed buildings or garbage incineration (Markus and McBratney, 2000). However, pollution by lead in the inhabited island is relatively low (i.e. most samples are below the limits allowed for agricultural soils; L.V. García, unpublished data) and neither affected body condition of amphisbaenians but only marginally for those individuals living directly under whitewhashed rocks that were a direct source of lead contamination (Martín *et al.*, 2015, 2017).

The PCA indicated that soils polluted by heavy metals also had lower pH values. Acidification of the soil, as a consequence of seagull depositions, may increase the bioavailability for plants of soil heavy metals (i.e. EDTA-extractable) that can, therefore, pass to the trophic chain (García *et al.*, 2007b; Jamali *et al.*, 2006). Nevertheless, a more acid pH *per se* might also affect amphisbaenians. In fact, a previous study showed that amphisbaenians avoided using the most acid soils (Martín *et al.*, 2013a), which could be partly related to the negative effects of soil acidity on the diversity and abundance of the invertebrates (Geissen *et al.*, 2007) that are prey of amphisbaenians (Martín *et al.*, 2013b).

In contrast, we have not found a relationship between GC levels (i.e. stress) and body condition of amphisbaenians. Similarly, in a previous study carried out in the same area (Martín *et al.*, 2015), we did not find an effect of soil heavy metals on amphisbaenians’ body condition. Body condition can be considered an index of nutritional state that should be mainly dependent on food availability, and in our study area this does not seem to be a limitation for amphisbaenians (Martín *et al.*, 2013b), even in contaminated soils. This suggests that several physiological measures should be considered to obtain a reliable general perspective of the health state of a population.

We also found an additional effect of the soil texture on the levels of faecal corticosterone metabolite of amphisbaenians. This is very likely related with the higher energetic costs of burrowing in harder substrates (i.e. those with less sand and more clay) (Gans, 1974; Dial *et al.*, 1987; Navas *et al.*, 2004). Sandy loose soils must be easier for burrowing, which would reduce energetic costs of foraging, behavioural thermoregulation, mate searching, etc. Similarly, in subterranean rodents, energetic costs of underground movement increase considerably in hard substrates (Seymour *et al.*, 1998; Luna and Antinuchi, 2006). In fact, soil texture is one of the main factors determining microhabitat selection in amphisbaenians (Martín *et al.*, 1991, 2013a), and other fossorial reptiles (Greenville and Dickman, 2009) and rodents (Jackson *et al.*, 2008). In addition, soil texture has a strong influence on retention and availability of water in the soil (i.e. water potential), which is lower in fine-textured soils with more clay (Fernández-Illescas *et al.*, 2001), especially in arid habitats, such as our study area. Thus, in soils with more clay, fossorial animals have low water availability and should be subjected to higher water losses, which must also increase GC levels during periods of drought.

Other soil properties described by the PCA reflecting soil alteration, such as eutrophication by birds and humans (PC-2; represented by high levels of P concentration) and salinization of marine origin (PC-4; represented by high Cl^−^ concentration) did not seem to affect faecal corticosterone metabolite levels of amphisbaenians in a significant way. However, alternatively, this might be simply explained because, on the one hand, the study islands show a more or less homogeneous distribution of highly eutrophicated soils throughout all their surfaces. Also, while soil salinity levels are known to affect body condition of amphisbaenians (Martín *et al.*, 2015), these animals seem to actively avoid using extreme saline soils (Martín *et al.*, 2013a, 2021a). Thus, even if these altered soils might also contribute to increase corticosterone levels of amphisbaenians, it would be difficult to find in our study area an enough large number of amphisbaenians living in soils with low eutrophication or high salinity with which to show clear relationships with GC levels.

Our study did not find differences in average faecal corticosterone metabolites levels between sexes or seasons. In contrast, in other animals it has been found, for example, a slightly higher levels of GCs in males during the breeding season, associated to costly reproductive behaviours (Dunlap and Wingfield, 1995; Romero, 2002; Moore and Jessop, 2003). The lack of differences in amphisbaenians in our study might initially suggest that changes in GC hormones are independent of sex and seasonal changes (the mating season occurs in spring) in reproductive hormones or behaviours. However, it could be more likely that the effect of environmental disturbances (soil alterations) increasing GC levels may be so strong that would mask any other potential sex or seasonal differences. Nevertheless, we did find clear differences between the two study years, suggesting that other environmental variables such as weather conditions (e.g. temperatures, rain, etc.) are also affecting to the levels of faecal corticosterone metabolite of amphisbaenians, as in other animals (Romero *et al*., 2000; Wingfield, 2013). Weather may affect animals either directly (e.g. thermal or hydric stress) or indirectly (e.g. through changes in prey availability or soil compaction). In fact, meteorological data showed that average temperatures in 2018 were slightly colder than in 2019 (19.1°C vs. 19.5°C) and total annual precipitations were also greater in 2018 (409 mm vs. 259 mm), but the summer dryness period was longer in 2018 (data from the Agencia Española de Meteorología). Future studies examining longer temporal series are, however, needed to eventually find the causes of a clear potential relationship between environmental temporal variation and GC levels.

Although physiological acute stress is an adaptive response to cope with stressful conditions, chronic long-term exposure to high GC levels can be very harmful (Moberg, 1999). Thus, one question that arises is what are the effects of the observed long-term increase in levels of GC in amphisbaenians for their health and fitness and, ultimately, for the dynamics and conservation of their populations. In other animals, stress chronic levels were associated with physiological damage, or immune inhibition, or suppression of reproduction (Dhabhar and Mcewen, 1997; Wingfield *et al.*, 1998; Sapolsky *et al.*, 2000; Ellenberg *et al.*, 2007; Meylan *et al.*, 2010). Therefore, future studies should examine and compare, for example, several physiological and reproductive parameters of the amphisbaenian populations inhabiting soils with more or less pollution.

Finally, we highlight that our study was made in a natural island reserve with ‘pristine’ conditions, where human access and activities are highly restricted. Thus, a direct human influence on these island ecosystems could *a priori* be considered negligible. However, our data show that human-derived pollution in the open sea and in coastal garbage dumps, where seagulls forage, can be indirectly and easily transferred by seagulls to the soils of the presumably protected reserve, with detrimental effects for soil animals.

We conclude that soil pollution by heavy metals and the variation in other soil characteristics, such as soil texture, may have strong negative effects on fossorial reptiles, which would be reflected in the observed increased levels of faecal corticosterone metabolite. Nevertheless, future experimental manipulations of different heavy metal contaminants and soil textures would be needed to confirm directionality in the relationships found in the current study. Soil pollution can have serious effects on health state and conservation of populations of fossorial animals, even in well-protected habitats, which may be unnoticed. Therefore, our study emphasizes that, to prevent future conservation problems of fossorial animals, we need to perform periodic surveys to collect baseline indicator data of the physiological health state of the little-known subterranean biodiversity. Moreover, future studies should try to better understand the characteristics of the stress response observed as well as the functional impacts on fitness relevant variables.

## Funding

This work was supported by the Spanish Ministerio de Ciencia, Innovación y Universidades project PGC2018–093592-B-I00 (MCIU/AEI/FEDER, UE).

## Data Availability

The datasets supporting the conclusions of this article are available in the Figshare repository (https://doi.org/10.6084/m9.figshare.16908493).

## Supplementary Material

supplementary-CONPHYS-2021-050_coab085
