# Supplementary material for: Soil pollution by heavy metals correlates with levels of faecal glucocorticoid metabolites of a fossorial amphisbaenian reptile
Source: Conserv Physiol. 2021 Nov 16;9(1):coab085. doi: 10.1093/conphys/coab085 (PMC8599815; doi:10.1093/conphys/coab085)
Supplement: supplementary-CONPHYS-2021-050_coab085 [file supplementary-conphys-2021-050_coab085.docx]

**Supplementary material**

**Table S1:** Set of best models (ΔAICc ≤ 4) for the effects of soil variables (total load of heavy metals, % sand, and concentrations of P and Cl^-^) and sampling year on faecal corticosterone metabolite levels of *T. wiegmanni* amphisbaenians.

|  | df | AICc | Likelihood ratio *χ^2^* | *P* |
| --- | --- | --- | --- | --- |
| Sand + Metals + Year | 3 | 368.66 | 50.30 | < 0.0001 |
| Metals + Year | 2 | 369.20 | 47.77 | < 0.0001 |
| P + Sand + Metals + Year | 4 | 370.27 | 50.69 | < 0.0001 |
| Sand + Cl + Metals + Year | 4 | 370.65 | 50.32 | < 0.0001 |
| Cl + Metals + Year | 3 | 371.08 | 47.88 | < 0.0001 |
| P + Metals + Year | 3 | 371.11 | 47.85 | < 0.0001 |
| P + Sand + Cl + Metals + Year | 5 | 371.84 | 51.12 | < 0.0001 |
